# Development and validation of a machine learning algorithm prediction for dense granule proteins in Apicomplexa

**DOI:** 10.1186/s13071-023-05698-0

**Published:** 2023-03-14

**Authors:** Zhenxiao Lu, Hang Hu, Yashan Song, Siyi Zhou, Olalekan Opeyemi Ayanniyi, Qianming Xu, Zhenyu Yue, Congshan Yang

**Affiliations:** College of Animal Science and Technology, School of Information and Computer, Anhui Agricultural University, Hefei, 230036 Anhui Province China

**Keywords:** Apicomplexa, Parasites, Machine learning, MVA-GCN, Dense granule protein

## Abstract

**Background:**

Apicomplexa consist of numerous pathogenic parasitic protistan genera that invade host cells and reside and replicate within the parasitophorous vacuole (PV). Through this interface, the parasite exchanges nutrients and affects transport and immune modulation. During the intracellular life-cycle, the specialized secretory organelles of the parasite secrete an array of proteins, among which dense granule proteins (GRAs) play a major role in the modification of the PV. Despite this important role of GRAs, a large number of potential GRAs remain unidentified in Apicomplexa.

**Methods:**

A multi-view attention graph convolutional network (MVA-GCN) prediction model with multiple features was constructed using a combination of machine learning and genomic datasets, and the prediction was performed on selected *Neospora caninum* protein data. The candidate GRAs were verified by a CRISPR/Cas9 gene editing system, and the complete *NcGRA64(a,b)* gene knockout strain was constructed and the phenotypes of the mutant were analyzed.

**Results:**

The MVA-GCN prediction model was used to screen *N. caninum* candidate GRAs, and two novel GRAs (NcGRA64a and NcGRA64b) were verified by gene endogenous tagging. Knockout of complete genes of *NcGRA64(a,b)* in *N. caninum* did not affect the parasite's growth and replication in vitro and virulence in vivo.

**Conclusions:**

Our study showcases the utility of the MVA-GCN deep learning model for mining Apicomplexa GRAs in genomic datasets, and the prediction model also has certain potential in mining other functional proteins of apicomplexan parasites.

**Graphical Abstract:**

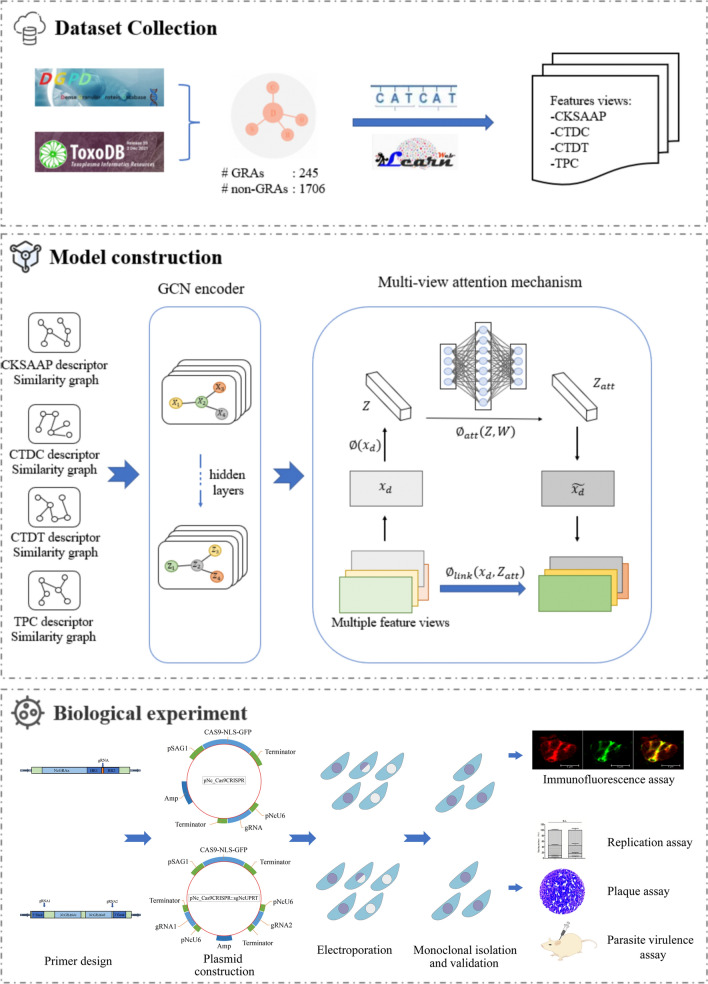

**Supplementary Information:**

The online version contains supplementary material available at 10.1186/s13071-023-05698-0.

## Background

Apicomplexa consist of numerous obligate intracellular protozoan organisms that cause severe parasitic diseases in humans, wildlife, and a variety of economically important livestock species, and include zoonotic parasites (*Plasmodium*, *Toxoplasma*, *Cryptosporidium*, *Babesia*) and other pathogenic parasites of livestock and wildlife (*Eimeria*, *Theileria*, *Neospora*) [1–4]. Apicomplexan parasites reside and replicate in parasitophorous vacuoles (PVs), which are modified by both parasite and host proteins that compartmentalize the parasite from the cytoplasm of the host cell, and through this interface the parasite nutrient exchange, effector transport, immune modulation and eventually egress occur [4, 5]. Previous studies have suggested that dense granules, which are specialized secretory organelles of the Apicomplexa, play a major role in the modification of PVs to maintain intracellular parasitism in host cells [6].

Dense granule proteins (GRAs) are a category of protein secreted by the dense granule organelle of the Apicomplexa and transferred to the PV, parasitophorous vacuole membrane (PVM), intravacuolar network, and the host cell cytoplasm or nucleus, which are closely related to the parasitism and development of the parasite in the cell. [7, 8]. The functions of these GRAs with different localization are also diverse, and include coordinating the vacuole building, modifying the vacuolar matrix and PVM, participating in tubular membrane formation, modulating host cell immune reactions, and affecting the transport of substances in the vacuolar membrane [7–9]. Although dozens of GRAs have been identified in the Apicomplexa model organism *Toxoplasma gondii*, a large number of potential GRAs remain unidentified in Apicomplexa.

To date, the discovery of GRAs has remained largely experimentally driven, ranging from traditional biochemical fractionation approaches to the latest proximity-dependent biotin identification (BioID) technique [5, 10–12]. Searching for novel GRAs through a large number of untargeted biological experiments is time-consuming and expensive. Hence, there is a need to develop a prediction tool to identify GRAs [11, 12]. Developments in sequencing technology have enabled the in-depth understanding of the Apicomplexa genome and provide new ideas for protein research, and bioinformatics methods are commonly used by current researchers to discover new functional proteins [13–15]. As a group of relatively small proteins, GRAs share some of the same characteristics, such as containing signal peptides, and most GRAs are predicted to be type I transmembrane proteins. These characteristics play an important role in GRA recognition [9]. Machine learning-based models have become popular in protein prediction recently. For instance, Zhang et al. explored the application of machine learning algorithms such as support vector machine (SVM) and artificial neural networks (ANNs) in predicting protein–protein interactions [16]. Huang et al. predicted the drug–target interaction (DTI) using an extremely randomized trees model and protein amino acid information [17]. However, with the development of next-generation sequencing (e.g., [14, 18]), traditional machine learning algorithms are less effective at handling complex and variable data. Deep learning is widely used in bioinformatics for the processing of data with multiple levels of abstraction (e.g., graph structure data with node features) [19, 20]. Notably, most models utilize single-type feature information for integrating multiple sources of information. In this paper, we employ an attention mechanism derived from the biological systems of humans to integrate multi-view to solve the above problem [21].

In a previous study, we constructed the first GRA database of the Apicomplexa, the Dense Granule Protein Database (DGPD) [22]. In the current study, we demonstrate that the multi-view attention graph convolutional network (MVA-GCN) combining multiple features can discover potential GRAs in Apicomplexa, and two novel *Neospora caninum* GRAs (NcGRA64a and NcGRA64b) were verified by biological experiments. Our current study highlights the potential of the combination of machine learning and genomic datasets to develop a GRA prediction tool and identify new functional GRAs.

## Methods

### Collection of the dataset

High quality of data is crucial for prediction models. To ensure the universality of the model, we first construct a GRA training set consisting of 245 positive samples and 1706 negative samples. The DGPD includes GRA information on some important apicomplexan parasites [22]. We retrieved 245 protein information including protein sequences from the above database as the positive samples, involving five important species of Apicomplexa (*Plasmodium falciparum*, *Toxoplasma gondii*, *Hammondia hammondi*, *N. caninum*, and *Cystoisospora suis*). The negative samples were collected from several protein databases, including ToxoDB (www.toxodb.org), PlasmoDB (www.plasmodb.org), Protein Data Bank (PDB; www.rcsb.org) and National Center for Biotechnology Information (NCBI; www.ncbi.nlm.nih.gov). To offset the impact of data imbalance, we collect the negative samples with similar species categories to positive samples to increase the training depth of the model. For preliminary collected negative samples, we performed a de-duplication against the different databases sources and removed all proteins with homology (cut-off E-value of 1 × 10^−5^) to the positive protein sample. For feature-based protein prediction models, common descriptors include multiple types of sequence encoding [23]. Therefore, we selected iLearn, a platform capable of calculating and extracting 18 major schemes of sequence coding, including 53 different types of protein sequence feature descriptors [24]. Multiple features were extracted from all samples by the iLearn tool. We finally selected four types of sequence descriptors, encompassing CKSAAP (composition of *k*-spaced amino acid pairs), CTDC (Composition/Transition/Distribution–Composition), CTDT (CTD–Transition), and TPC (tri-peptide composition), as the sample features. The ratio of positive to negative samples was maintained at 1:8.

### Construction of graphs

To meet the experimental requirements, we constructed graphs for different features, respectively. The *k*-nearest neighbor (KNN) graph is a common graph structure in machine learning [25]. In different GRA feature matrixes, we find the *k* value of the most similar neighbors for each sample point using the Minkowski distance. The formula is as follows:

$$dis = \left( {\sum\nolimits_{{k = 1}}^{n} {\left| {P_{k} \, - \,Q_{k} } \right|^{r} } } \right)^{{\frac{1}{r}}}$$

where *r* is a variable parameter that indicates various Minkowski distances, *n* is the dimension (property) number of the feature, and ${P}_{k}$ and ${Q}_{k}$ are the *k*th dimension of data objects *P* and *Q*, respectively. The parameter *r* is usually set to 2.

We obtained the adjacency matrix of samples set from the KNN graph and obtained the nodes embedded in the feature matrix. Deep Graph Library (DGL) is an effective, graph-centric open-source Python package for graph convolution networks [26]. We utilized the KNN graph obtained above and the Python package to construct an input for our model. Notably, in the process of KNN graph construction, the number (*k*) of neighbors of each node is defined in $\left\{3, 5, 7\right\}$ for exploring the effect of the neighbor amount in graphs on the model. For detailed analysis, see the “Development and evaluation of the model” section.

### Creation of GCN encoder

The MVA-GCN was created in modular fashion. Firstly, the feature views were constructed by the method in the “Construction of graph” section. Subsequently, for capturing information of multiple views, we constructed a GCN encoder to encode multiple views. The GCN encoder provides a graph-based neural network $f\left(X, A\right)$, and we considered a layer-wise propagation approach for the GCN model:

$${H}^{\left(l+1\right)} = \sigma \left({\widetilde{D}}^{-\frac{1}{2}}\widetilde{A}{\widetilde{D}}^{-\frac{1}{2}}{H}^{\left(l\right)}{W}^{\left(l\right)}\right).$$

Here, $\widetilde{A} =A +I$, $I$ is the identity matrix, $\tilde{D}\, = \,\sum\nolimits_{j} {\mathop {{\text{A}}_{ij} }\limits^{ \vee } }$ is the degree matrix of $\widetilde{A}$, and ${W}^{\left(l\right)}$ is a trainable weight matrix of the specific layer. ${H}^{\left(l\right)}$ is an activation matrix in the ${l}^{th}$ layer, and corresponds to the input layer ${H}^{\left(0\right)} =X$. $\sigma \left(\bullet \right)$ is an activation function, and two GCN layers all adopt a rectified linear unit (ReLU) function. Therefore, the propagation formula of the model is as follows:

$$Z=f\left(X, A\right) =ReLU\left(\widetilde{A} ReLU\left(\widetilde{A}X{W}^{\left(0\right)}\right){W}^{\left(1\right)}\right).$$

Here, *X* is a matrix composed of node features in the graph, and *A* is the adjacency matrix of the graph. Then, we compute the cross-entropy loss for all labeled nodes:

$$\mathcal{L}=-\sum_{l\in {y}_{L}}\sum_{f=1}^{F}{Y}_{lf}\mathrm{ln}{Z}_{lf},$$

where ${y}_{L}$ is an indicator set of labeled nodes.

### Multi-view attention mechanism

Single-view embedding may lead to results that are less than expected. Therefore, we constructed a multi-view attention mechanism to give different contributions for model embeddings.

We made horizontal connections on the matrices of multiple views and obtained the statistics based on the views using global average pooling. A *Z*-statistic was used to calculate the view importance. For the matrix ${x}_{d}$ of the protein *d*th view, the statistic ${Z}_{d}$ was calculated as follows:

$${Z}_{d}= \varnothing \left({x}_{d}\right)= \frac{1}{N\times M}\sum_{i=1}^{N}\sum_{j=1}^{M}{x}_{d}\left(i,j\right).$$

To capture the importance of multiple features, we utilized an attention mechanism to calculate the attention weights of different views:

$${Z}_{att}= {\varnothing }_{att}\left(Z,W\right)= \delta \left({W}_{2}\sigma \left({W}_{1}Z\right)\right).$$

Here, $\delta$ represents the sigmoid activation,$\sigma$ is ReLU activation, and $W=\left\{{W}_{1},{W}_{2}\right\}$ is the trainable parameter. Finally, we obtain the multi-view attention ${Z}_{att}=\left[{Z}_{1}^{att},{Z}_{2}^{att},\cdots ,{Z}_{T}^{att}\right]$. We consider feature views and attention together. The formula is as follows:

$$\widetilde{{x}_{d}}={\varnothing }_{link}\left({x}_{d},{Z}_{d}^{att}\right)={Z}_{d}^{att}\bullet {x}_{d}.$$

Up to this point, we have obtained the standardized protein information $\widetilde{X}=\left[\widetilde{{x}_{1}},\widetilde{{x}_{2}},\cdots ,\widetilde{{x}_{T}}\right]$ as the final embedding.

There are general complex linear relationships in the node features. Hence, we utilize a convolutional neural network (CNN) to process the above results and integrate the outputs of multiple-view embedding to generate the final output.

### Parasites and cell cultures

African green monkey kidney (Vero) and human foreskin fibroblast (HFF) cell lines were cultured in a humidified incubator at 37 °C and 5% CO_2_ using Dulbecco's modified Eagle’s medium (DMEM, Gibco, USA) supplemented with 10% fetal bovine serum (FBS, Gibco, USA) and 1% penicillin and streptomycin [11, 27]. The parental and mutant strains in the tachyzoite form of *N. caninum* were maintained in vitro by serial passage on confluent Vero cells.

### Plasmid construction

All primers used for cloning and genetic manipulation sequences can be found in Additional file 1: Table S1. To generate endogenously tagged strains of *NcGRA64(a,b)* genes, CRISPR plasmids pNc_Cas9CRISPR::sgNcGRA64a and pNc_Cas9CRISPR::sgNcGRA64b targeting *NcGRA64a* and *NcGRA64b*, respectively, were constructed as described previously [27]. The spaghetti monster-HA (smHA) tag in the psmHA-DHFR vector plasmid was used for in situ C-terminal tagging, as described previously [11]. To generate a clean knockout strain of *NcGRA64(a,b)* genes, a double-guide RNA (gRNA) CRISPR/Cas9 system was constructed, in which the first gRNA sequence (gRNA1) was placed close to the start codon of the *NcGRA64a* gene nearby, and a second gRNA (gRNA2) was located near the stop codon of the *NcGRA64b* gene, as described previously [11]. The gRNA2 expression cassette was amplified using a common set of primers 2 × gRNA NcU6 F/R and pNc_Cas9CRISPR::sgNcGRA64(a,b)^2^ plasmid as template, and cloned into pNc_Cas9CRISPR::sgNcGRA64(a,b)^1^ plasmid using a one-step cloning kit (Vazyme Biotech Co., Ltd., China) to obtain pNc_Cas9CRISPR::sgNcGRA64(a,b) plasmid. For disruption of *NcGRA64(a,b)* genes, CRISPR/Cas9 double-gRNA plasmid pNc_Cas9CRISPR::sgNcGRA64(a,b) was co-transfected with its corresponding DHFR-TS amplicon containing 60-base-pair (bp) homology regions matching the *NcGRA64(a,b)* genes.

### Construction of endogenously tagged strain

The primers listed in Additional file 1: Table S1 and the psmHA-DHFR plasmid were used as a template to amplify the smHA-tagged amplicon containing 42-bp homology regions matching the gene of *NcGRA64a* and *NcGRA64b*, respectively. The pNc_Cas9CRISPR::sgNcGRA64a/sgNcGRA64b plasmids and their corresponding smHA-tagged amplicons were co-transfected into Nc1 parasites by electroporation. Selection of transfected parasites was performed 24 h post-transfection with a medium containing 1 μM pyrimethamine, identified by polymerase chain reaction (PCR) and immunofluorescence assay (IFA).

### Construction of gene knockout strain

The DHFR-TS amplicon containing a 60-bp homology region matching the genes of *NcGRA64(a,b)* was amplified using the primers listed in Additional file 1: Table S1 [NcGRA64(a,b)-KO-F/R] as previously described [11]. The pNc_Cas9CRISPR::sgNcGRA64(a,b) plasmid and dihydrofolate reductase–thymidylate synthase (DHFR-TS) amplicons were co-transfected into Nc1 parasites by electroporation. Selection of transfected parasites was performed 24 h post-transfection with a medium containing 1 μM pyrimethamine, and single clones were obtained by limiting dilution and identified by PCR. The selected *NcGRA64(a,b)* knockout strain was designated as Δ*ncgra64(a,b)*.

### Immunofluorescence assay

HFFs were seeded on coverslips in 12-well plates (Nest, China) and allowed to form confluent monolayers, which were then infected using 1 × 10^4^ tachyzoites and incubated at 37 °C with 5% CO_2_ for 30 h, as described previously [27, 28]. All coverslips were fixed with 4% paraformaldehyde for 30 min at room temperature and then permeabilized in a 0.25% Triton X-100 solution for 15 min at room temperature, rinsed with phosphate-buffered saline (PBS), and blocked with 3% bovine serum albumin (BSA) for either 45 min at room temperature or 4 °C overnight. Subsequently, the cells were incubated with mouse anti-NcGRA6 polyclonal antibody (1:50, prepared in our laboratory) and rabbit anti-HA polyclonal antibody (CWBIOTECH, China, 1:50) at 37 °C for 1 h, followed by PBS washes (3 × 5 min). Incubation was then performed with fluorescein isothiocyanate (FITC)-conjugated goat-anti mouse immunoglobulin G (IgG) and cyanine 3 (Cy3)-conjugated goat-anti-rabbit IgG (1:100, Proteintech, USA) at 37 °C for 1 h, followed by final PBS washes (3 × 5 min). Coverslips were mounted on slides supplemented with Fluoromount-G (Macgene, China) and imaged using a Leica confocal microscope system (Olympus Co., Japan).

### Plaque assay

HFFs were seeded in 12-well plates (Nest, China) as described previously [27, 28], and allowed to form confluent monolayers. A total of 200 freshly egressed parasites were seeded into HFF monolayers and incubated at 37 °C with 5% CO_2_ for 9 days undisturbed, after which the medium was discarded and the plates were thoroughly washed three times with PBS. They were then fixed with 4% paraformaldehyde for 30 min at room temperature and finally stained with 1% crystal violet for 30 min at room temperature. After thorough washing with PBS, the plaque area was measured by scanning with a Canon digital scanner (model F917500, Japan) [29].

### Replication assay

HFFs were seeded in 12-well plates (Nest, China) and allowed to form confluent monolayers. Next, 1 × 10^6^ tachyzoites from parent Nc1 or Δ*ncgra64(a,b)* strains were seeded into HFF monolayers and incubated for 30 min, followed by three washes with PBS to remove unbound parasites, and were then incubated at 37 °C with 5% CO_2_ for 30 h and subsequently fixed using 4% paraformaldehyde. IFAs followed using rabbit anti-NcSRS2 polyclonal antibody (1:500, prepared in our laboratory) and Cy3-conjugated goat-anti-rabbit IgG (1:100, Proteintech, USA) antibodies. The number of parasites per vacuole was counted for each strain using a fluorescence microscope (Olympus Co., Japan). The experiment was repeated three times independently, and a total of 200 PVs were counted for each strain in each replicate. Statistical analysis was performed with the Chi-square test using SAS software (SAS Institute Inc., USA). Differences were considered significant for values of *P* ≤ 0.05.

### Parasite virulence assay

Female, 6-week-old BALB/c mice purchased from the Laboratory Animal Center of Anhui Medical University (Hefei, China) were randomly assigned to five mice per test group for the virulence experiments. Each mouse group was intraperitoneally injected with 5 × 10^6^ or 8 × 10^6^ tachyzoites from the parent Nc1 or Δ*ncgra64(a,b)* strains. The clinical signs and mortality of the mice were observed and recorded daily for 60 days. The mice were humanely euthanized via cervical dislocation when they were unable to reach food or water for more than 24 h or lost 20% of normal body weight.

## Results

### Development and evaluation of the model

To evaluate the performance of our model, we divided the dataset into a training set and an independent test set at a ratio of 7:3 and used them for multiple rounds of testing. We take into account the importance of the parameter sensitivity of MVA-GCN, including the number of nearest neighbors, the layers of GCN, and the filter size. In each round, we selected the layers of the GCN encoder from $\left\{2, 3, 4\right\}$, the number of nearest neighbors was selected in $\left\{3, 5, 7, 9\right\}$, and the learning rate was selected from $\left\{0.1, 0.01, 0.001\right\}$. We analyzed other related projects and set the dropout rate for all layers to 0.7 [30, 31], and the number of GCN hidden units was fixed to 256 [32]. We trained the model on the constructed dataset and comprehensively evaluated the model with results from the independent test set.

We calculated the common evaluation metrics including the area under the precision–recall curve (AUPRC) [33], area under the ROC curve (AUC) [34], accuracy, precision, recall, and F1-score. In binary classification problems, researchers prefer to use AUPRC and AUC [35]. For the requirements of the task, exploring GRAs with the pre-ranked result from the computational model, we adopted AUPRC and precision as the primary metrics during experiments.

### Comparison with other methods

We compared multiple single-view GCN models, and used four types of protein feature descriptors as the model input, including CKSAAP, CTDC, CTDT, and TPC. Simultaneously, the MVA-GCN in this study performs multiple experiments of GRA prediction with the parameters described in the “Development and evaluation of the model” section. Finally, we constructed two layers of GCN; the number of hidden layers was 256, the learning rate and dropout value were set at 0.01 and 0.7, respectively, the number of nearest neighbors was 3, and the learning rate was determined as 0.1. The result is shown in Table 1. The use of multiple features can improve the accuracy of predictions. Targeting existing GCN models with single-feature view, MVA-GCN combines multiple features and fuses view information by the attention mechanism. The performance of MVA-GCN was significantly improved for GCN models with single-feature view. The AUPRC and precision were maintained at 0.9487 and 1 on the test set. The experimental results indicate that the MVA-GCN that we constructed was more efficient in identifying novel GRAs.

**Table 1:** Comparison of performance

Method	AUPRC	Precision	Accuracy	AUC	F1	Recall	Specificity
MVA-GCN	0.9487^a^	1.0000^a^	0.9658^b^	0.9673^a^	0.8181^b^	0.6923^b^	1.0000^a^
GCN-CKSAAP	0.8456^b^	0.9250^b^	0.9508	0.9508^b^	0.7531	0.6388	0.9929^b^
GCN-CTDC	0.8071	0.9032	0.9622	0.8885	0.8115	0.6855	0.9893
GCN-CTDT	0.8310	0.9113	0.9663^a^	0.9113	0.8372^a^	0.7230^a^	0.9856
GCN-TPC	0.7788	0.8541	0.9343	0.9268	0.6578	0.5413	0.9870

We calculated each of the AUPRC, AUC, accuracy, precision, recall, and F1-score models. MVA-GCN had the highest precision, followed by AUPRC

^a^Highest value of each indicator

^b^Second-best value of each indicator

### Experimental validation of novel dense granule proteins

We mined massive protein data of apicomplexan parasites, and hundreds of protein sequences of *Toxoplasma* and *Neospora* were screened out randomly. We utilized MVA-GCN to perform prediction on collated protein data, and the probabilities of proteins belonging to GRAs were obtained by the model return. After analysis, we found two proteins NCLIV_022320 and NCLIV_022330 with a probability greater than 0.5 (Additional file 2: Table S2). Sequence analysis revealed that they shared extensive sequence similarity. To verify the accuracy of the results, we conducted biological experiments.

**Fig. 1:**
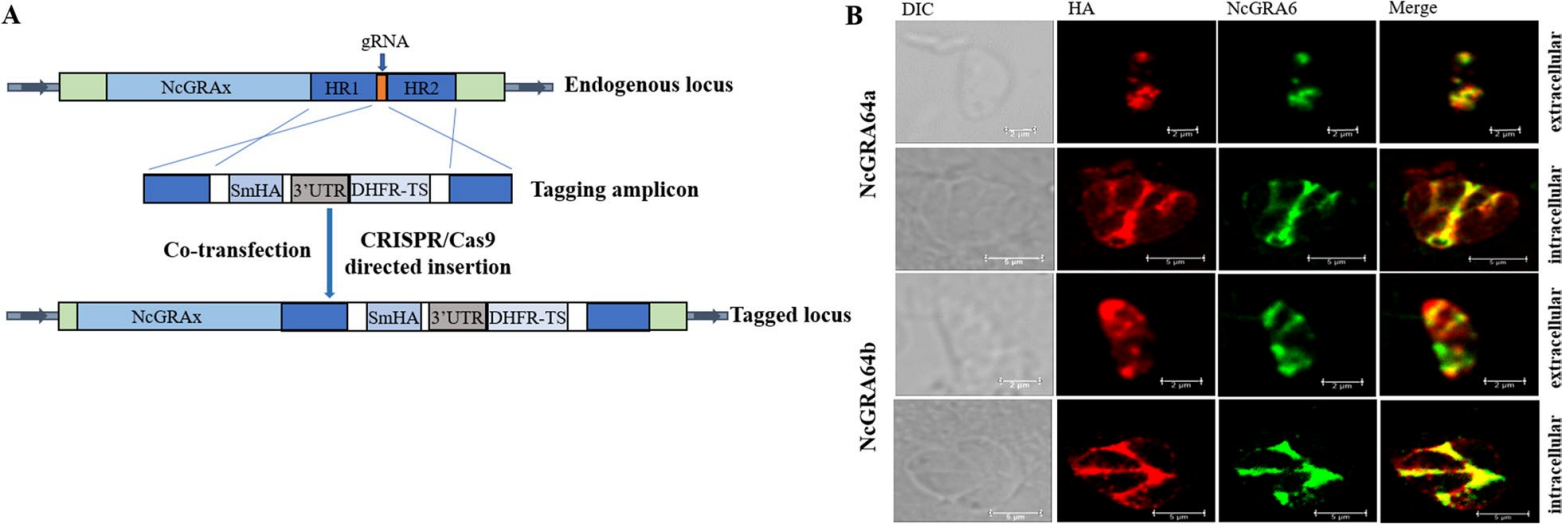
Identification of novel dense granule proteins. **A** Schematic illustration of endogenous gene tagging at the C-terminus by CRISPR/Cas9-mediated site-specific insertion. **B** IFA results showing that HA-tagged NCLIV_022320 and NCLIV_022330 co-localize with NcGRA6 in dense granules (intracellular, scale bar = 5 μm; extracellular, scale bar = 2 μm)

To examine the subcellular localization of these two proteins, we used CRISPR/Cas9 to add HA epitope tags to the C-termini, respectively (Fig. 1A). IFA analysis of the endogenously tagged strain indicated that the NCLIV_022320 and NCLIV_022330 proteins of the extracellular parasite co-localize with the dense granule marker NcGRA6; the HA tag of the intracellular tachyzoites was localized on the PV (Fig. 1B). These findings indicate that the proteins encoded by the *NCLIV_022320* and *NCLIV_022330* genes are GRAs. Therefore, NCLIV_022320 and NCLIV_022330 were designated as NcGRA64a and NcGRA64b, respectively.

### Functional characterization of *NcGRA64(a,b)*

**Fig. 2:**
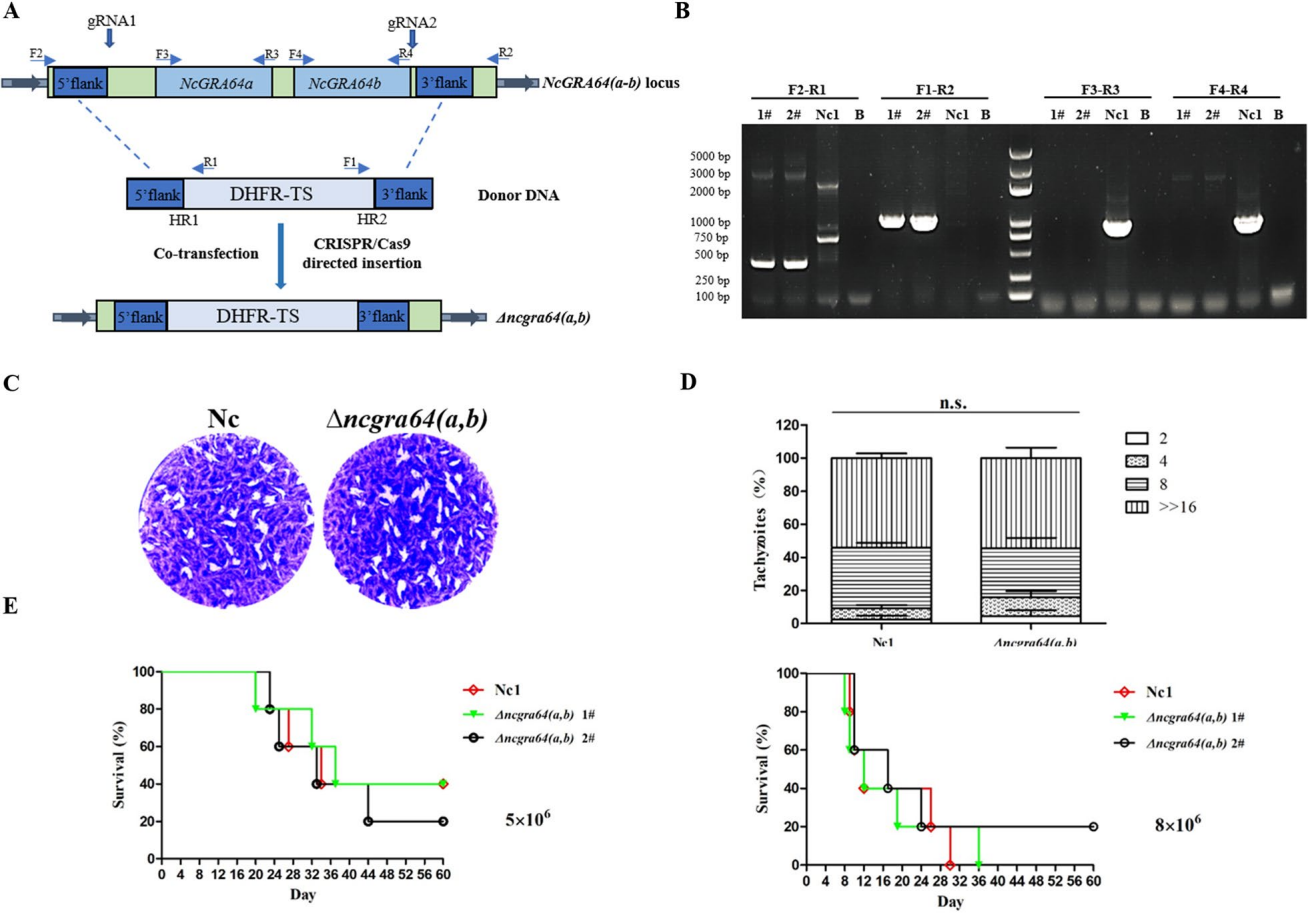
Functional characterization of NcGRA64(a,b). **A** Schematic illustration of the disruption of *NcGRA64(a,b)* by CRISPR/Cas9-mediated homologous gene replacement. **B** Diagnostic PCRs on two Δ*ncgra64(a,b)* clones (1 and 2). (F2-R1) and (F1-R2) check the correct integration of the selection marker to the *NcGRA64(a,b)* genes locus, whereas (F3-R3) and (F4-R4) examine the deletion of the *NcGRA64(a,b)* genes. **C** Plaque assay comparing the growth of parent Nc1 and Δ*ncgra64(a,b)* strains in vitro. Purified tachyzoites were used to infect HFF monolayers (200/well) seeded in 12-well plates, and plaques were stained 9 days later. **D** Intracellular parasite replication of Nc1 and Δ*ncgra64(a,b)* strains. Data were compiled from three independent assays, and a total of 200 PVs of each strain were counted in each assay. Data were analyzed using the Chi-square test. **E** Mouse survival after infection with 5 × 10^6^ and 8 × 10^6^ doses of Nc1 or Δ*ncgra64(a,b)* strains. BALB/c mice were infected with tachyzoites from Nc1 or Δ*ncgra64(a,b)* strains by intraperitoneal injection, and the survival of the mice was monitored daily. Statistical analysis was performed using the life test in a statistical analysis system (SAS Institute Inc., USA). The data are representative of two experiments with similar outcomes

The *NCLIV_022320* and *NCLIV_022330* genes encoding two novel GRAs identified in this study were homologous genes, and it was verified that these two genes were located together on chromosome 7 for 5100 bp (Fig. 2A). To verify the biological role of the two genes, we constructed a clean *NcGRA64(a,b)* genes knockout strain Δ*ncgra64(a,b)* using double-gRNA targeting the 5′ and 3′ regions of the sequence to delete the entire sequence, followed by selection for insertion of the DHFR-TS selectable maker, diagnostic PCR confirmed the deletion of the *NcGRA64(a,b)* genes (Fig. 2B). We assessed the Δ*ncgra64(a,b)* strains using the plaque and replication assays. The results indicated that *NcGRA64(a,b)* were not involved in parasite growth and replication, as the plaque area of Δ*ncgra64(a,b)* was equivalent to that of parent Nc1 (Fig. 2C), and the intracellular replication ability of Δ*ncgra64(a,b)* was not significantly different from that of Nc1 (Fig. 2D). To evaluate the function of *NcGRA64(a,b)* in vivo, the 5 × 10^6^ or 8 × 10^6^ tachyzoites of the different strains were used to infect mice by intraperitoneal injection and test their virulence. We observed no significant difference in survival time between mice inoculated with parent Nc1 or Δ*ncgra64(a,b)* strains(Fig. 2E), indicating that these proteins do not affect parasite virulence in mice.

## Discussion

Dense granules are specialized secretory organelles of apicomplexan parasites. GRAs released from dense granules are thought to play important roles in both protein and lipid trafficking events induced by parasites [9]. A deeper understanding of dense granules will help us explore the pathogenesis of Apicomplexa. However, currently, the experimentally validated GRAs account for only a fraction of all GRAs in apicomplexan parasites, and the full proteome of dense granules remains unknown. Recently, the BioID technique has become widely used for GRA screening, but its application is limited due to its poor temporal resolution as a result of low catalytic activity [10, 11, 36, 37]. Hence, it is necessary to develop more convenient tools for GRA screening.

With the development of deep learning, machine learning has been applied more widely in the research of apicomplexan parasites, including the prediction of antimalarial drugs and parasite detection [38–41]. At the same time, many new computing methods have been proposed and used in the discovery of new substances in living things. For example, GCNs are usually used for graph-structured data in bioinformatics [32]. In this study, we constructed a prediction model MVA-GCN for the identification of novel GRAs. Notably, in previous experiments, researchers have preferred to utilize GCN with single-type feature information for prediction tasks. Our study shows that MVA-GCN with multiple features information has better efficiency in GRA prediction. Furthermore, as an important branch of the protein prediction field, machine learning plays a non-negligible role. Hence, we additionally used traditional algorithms, including decision trees (DT), random forest (RF), and SVM. Herein, the performance of the SVM is consistently superior to other traditional models, but the comparison with our model is inferior (Table 2). The average AUPRC and precision of the SVM are 0.8078 and 0.7815, respectively. MVA-GCN is the first deep learning model to predict novel GRAs, and has extensive application prospects. Follow-up studies confirmed that our method can discover various unreported GRAs. The application of this study greatly promotes the identification and prioritization of GRAs, and helps experimenters to explore more novel parasite-specific drug targets for related diseases.

**Table 2:** Comparison of traditional algorithms

Method	AUPRC	Precision	Accuracy	AUC	F1	Recall	Specificity
MVA-GCN	0.9088^a^	1.0000^a^	0.9658^a^	0.9673^a^	0.8181^a^	0.6923^a^	1.0000^a^
SVM	0.7815^b^	0.8078^b^	0.9369^b^	0.6943	0.7531^b^	0.6117^b^	0.9800
RF	0.3639	0.5148	0.8831	0.8069^b^	0.1306	0.0771	0.9912^b^
DT	0.5360	0.5823	0.9343	0.7015	0.4996	0.4409	0.9553

^a^Highest value of each indicator

^b^Second-best value of each indicator

We eventually selected two GRA candidates from the predicted data, and further experiments were conducted to examine their subcellular localization and functional characterization. We first prepared mouse anti-NcGRA64a and anti-NcGRA64b polyclonal antibodies. However, the serum we prepared did not bind to the corresponding protein in IFA, which may be due to its weak antigenic determinant binding ability and other factors. Then we used a CRISPR/Cas9 gene editing system to tag endogenous markers in the Nc1 background, as previously described in *Neospora* [11]. IFA analysis indicated that NcGRA64a and NcGRA64b co-located with NcGRA6 with the dense granules and at the PV. We found that *NcGRA64a* and *NcGRA64b* genes shared extensive sequence similarity and were located together on chromosome 7 for 5100 bp by sequence analysis. CRISPR/Cas9 double-gRNA plasmid was used to construct deletions of the genes encoding *NcGRA64a* and *NcGRA64b*, as described previously [11]. Like most GRAs, the knockdown of complete genes of *NcGRA64(a,b)* in the *N. caninum* strain did not affect the parasite's growth and replication in vitro and did not affect its virulence in the process of infection in mice. However, this does not preclude the possibility that NcGRA64a and NcGRA64b have other important biological roles.

## Conclusions

Our study showcases the utility of combining the MVA-GCN deep learning model and genomic datasets for the mining of GRAs in Apicomplexa. Compared with the methods based on biological experiments, the MVA-GCN deep learning model has higher accuracy and time-saving procedures. In addition, we believe that the MVA-GCN deep learning model has certain potential in mining other functional proteins of apicomplexan parasites.

## Supplementary Information

**Additional file 1: Table S1.** Primers used in this study.

**Additional file 2: Table S2.** Predictive results of case study.

## Data Availability

All data analyzed or generated during this study are included in this published article.

## Abbreviations

*N. caninum*: *Neospora caninum*
GRA: Dense granule protein
MVA-GCN: Multi-view attention graph convolutional network
NcGRA: *Neospora* dense granule protein
PV: Parasitophorous vacuole
BioID: Biotin identification
PVM: Parasitophorous vacuole membrane
DMEM: Dulbecco’s modified Eagle’s medium
Vero: African green monkey kidney cell
HFF: Human foreskin fibroblast
DT: Decision tree
RF: Random forest
SVM: Support vector machine
ANNs: Artificial neural networks
CKSAAP: Composition of *k*-spaced amino acid pairs
TPC: Tri-peptide composition
