# Supplementary material for: Development and validation of a machine learning algorithm prediction for dense granule proteins in Apicomplexa
Source: Parasit Vectors. 2023 Mar 14;16:98. doi: 10.1186/s13071-023-05698-0 (PMC10012559; doi:10.1186/s13071-023-05698-0)
Supplement: Supplementary file 1 — Additional file 1: Table S1. Primers used in this study. [file 13071_2023_5698_MOESM1_ESM.docx]

Development and validation of a machine learning algorithm prediction for dense granule proteins for Apicomplexa

Zhenxiao Lu^1†^, Hang Hu ^1†^, Yashan Song^1^, Siyi Zhou^1^, Ayanniyi Olalekan Opeyemi^1^, Qianming Xu^1^, Zhenyu Yue^1^* and Congshan Yang^1^*

Table S1. Primers used in this study.

| Primer name | Sequences |
| --- | --- |
| Nc5-F | 5′- CAGTCAACCTACGTCTTCT -3′ |
| Nc5-R | 5′- GTGCGTCCAATCCTGTAAC -3′ |
| R1 | 5′- TTGCGGTGTCGTGGATTTAC -3′ |
| F1 | 5′- ACAGTCTCACCTCGCCTTGT -3′ |
| F2 | 5′- TAAGCAGCGTTACCGCCCTG -3′ |
| R2 | 5′- CTCCACAATGGCGGTGGTTC -3′ |
| F3 | 5′- ATGAGGCTATTTTGCCCGGTCT -3′ |
| R3 | 5′- AGAACCGTCTGTATCTCTCAC -3′ |
| F4 | 5′- ATGAGGCTATTTTGCCCGGTCT -3′ |
| R4 | 5′- AGGAACGTATGTGCCCCTCAC -3′ |
| Cas9 F | 5′- ATACGACTCACTATAGGGCG -3′ |
| Cas9 R | 5′- AGCTCCACCGCGGTGGCGGC -3′ |
| NcU6-gRNA F | 5′- GCCGCCACCGCGGTGGAGCTGAGGGCAAAACTCCACAAA -3′ |
| gRNA-AMP R | 5′- CGCCCTATAGTGAGTCG -3′ |
| NcGRA64a-gRNA-AMP-F | 5′- GTGCGCCCGAATGCTCTGTGGTTTTAGAGCTAGAAATAG-3′ |
| NcU6-NcGRA64a-gRNA-R | 5′- CACAGAGCATTCGGGCGCACAAACAACAATGTCCCTTTG-3′ |
| NcGRA64b-gRNA-AMP-F | 5′- GCGTCCCTGTACGCCCGTATGTTTTAGAGCTAGAAATAG-3′ |
| NcU6-NcGRA64b-gRNA-R | 5′- ATACGGGCGTACAGGGACGCAAACAACAATGTCCCTTTG-3′ |
| NcU61-NcGRA64(a,b)_gRNA1-R | 5′- CCCCTCATAGAGCGTCAAGGAAACAACAATGTCCCTTTG-3′ |
| NcGRA64(a,b)_gRNA1-AMP-F | 5′- CCTTGACGCTCTATGAGGGGGTTTTAGAGCTAGAAATAG-3′ |
| 2×gRNA backbone F1 | 5′- CGAGGTCGACGGTATCGATA -3′ |
| 2×gRNA backbone R1 | 5′- AGCTCCACCGCGGTGGCGGC -3′ |
| 2×gRNA backbone F2 | 5′- GCCGCCACCGCGGTGGAGCT -3′ |
| 2×gRNA backbone R2 | 5′- CGCCCTATAGTGAGTCG -3′ |
| 2×gRNA NcU6 F | 5′- TACGACTCACTATAGGGCGGAGGGCAAAACTCCACAAAA -3′ |
| 2×gRNA NcU6 R | 5′- TATCGATACCGTCGACCTCGCACAGGAAACAGCTATGAC -3′ |
| NcGRA64a-HR1-F | 5′- AAAGCTCAGCGGCGTGCGGTTGTGAGAGATACAGACGGTTCTGCTAGCAAGGGCTCGG-3′ |
| NcGRA64a-HR2-R | 5′- CTATCAAGGTGGAAGGTAGCAAGCGACGGTTTGCTCCCACACATACGACTCACTATAGG-3′ |
| NcGRA64b-HR1-F | 5′- AAAGCTCAGCGACGTGCGGTGGTGAGGGGCACATACGTTCCTGCTAGCAAGGGCTCGG-3′ |
| NcGRA64b-HR2-R | 5′- TCCCCCAGGCTTCTGTCAGAACGTCACATCGAATACCCGATAATACGACTCACTATAGG-3′ |
| NcGRA64(a,b)-KO-F | 5′- GCTGTCTGGGACTCGTTTCACGTGGCTTCCCATTTGGTTTTC GCCTTGACGCTCTATGAGCTAGCATGTCATTCGATTTT-3′ |
| NcGRA64(a,b)-KO-R | 5′- AACTACGTCACCATCAAATCCCCCAGGCTTCTGTCAGAACGTCAC ATCGAATACCCGATAACTAGTGGATCGATCCCCCG-3′ |
|  |  |
